# Temporal Lobe Resective Surgery for Medically Intractable Epilepsy: A Review of Complications and Side Effects

**DOI:** 10.1155/2013/752195

**Published:** 2013-10-31

**Authors:** Iordanis Georgiadis, Effie Z. Kapsalaki, Kostas N. Fountas

**Affiliations:** ^1^Departments of Neurosurgery, University Hospital of Larisa, Faculty of Medicine, University of Thessaly, Biopolis, Larissa 41110, Greece; ^2^Departments of Neurosurgery & Diagnostic Radiology, University Hospital of Larisa, Faculty of Medicine, University of Thessaly, Larissa, Greece; ^3^CERETETH, Center for Research and Technology of Thessaly, Larissa 38500, Greece

## Abstract

*Object*. It is widely accepted that temporal resective surgery represents an efficacious treatment option for patients with epilepsy of temporal origin. The meticulous knowledge of the potential complications, associated with temporal resective procedures, is of paramount importance. In our current study, we attempt to review the pertinent literature for summating the complications of temporal resective procedures for epilepsy. *Method*. A PubMed search was performed with the following terms: “behavioral,” “cognitive,” “complication,” “deficit,” “disorder,” “epilepsy,” “hemianopia,” “hemianopsia,” “hemorrhage,” “lobectomy,” “medial,” “memory,” “mesial,” “neurobehavioral,” “neurocognitive,” “neuropsychological,” “psychological,” “psychiatric,” “quadranopia,” “quadranopsia,” “resective,” “side effect,” “surgery,” “temporal,” “temporal lobe,” and “visual field.” *Results*. There were six pediatric, three mixed-population, and eleven adult surgical series examining the incidence rates of procedure-related complications. The reported mortality rates varied between 0% and 3.5%, although the vast majority of the published series reported no mortality. The cumulative morbidity rates ranged between 3.2% and 88%. *Conclusions*. Temporal resective surgery for epilepsy is a safe treatment modality. The reported morbidity rates demonstrate a wide variation. Accurate detection and frank reporting of any surgical, neurological, cognitive, and/or psychological complications are of paramount importance for maximizing the safety and improving the patients' overall outcome.

## 1. Introduction

It is well known that epilepsy constitutes one of the most common neurological clinico-pathological entities, affecting approximately 1% of the general population [1]. It has been estimated, that its prevalence in North America varies between 5 and 10 per 1000 people, and it affects people from all races, ethnicities, and socioeconomic backgrounds [1]. Therefore, epilepsy represents a common clinical condition with significant medical sequences but also serious social and economic ramifications. It has been demonstrated that temporal lobe epilepsy represents by far the most common form of focal epilepsy in adults, while it is one of the most common forms of epilepsy in children [2–7]. Temporal lobe epilepsy usually presents with simple and/or complex partial seizures, although the underlying pathology may be any of a wide spectrum of pathological entities, such as hippocampal sclerosis, low grade glial tumors (dysembryoplastic neuroepithelial tumor, ganglioglioma, and oligodendroglioma), neuronal migrational disorders (cortical dysplasia), and vascular lesions (cavernous malformation and arteriovenous malformation), while in a significant number of cases no structural abnormalities can be found despite the exhausting imaging workup.

A large number of clinical and epidemiological studies have shown that approximately a third of adult patients suffering from epilepsy will eventually develop medically refractory epilepsy, despite proper administration of the indicated anticonvulsants [1, 7, 8]. Similarly, approximately 10–20% of children with epilepsy will develop, at some point, medically refractory epilepsy [9–11]. It has been adequately demonstrated, that surgical treatment of patients with medically intractable epilepsy is the method of choice for managing them, particularly in cases of temporal lobe epilepsy [1, 12, 13]. Wiebe et al. in prospective, randomized, and controlled studies have clearly shown that surgical treatment of patients with epilepsy of temporal origin is superior to any kind of medical treatment [1]. Besides, it offers to those patients a good chance to become seizure-free, while it significantly improves their quality of life. Despite the rapidly growing body of evidence regarding the efficacy of epilepsy surgery in cases of temporal epilepsy, the number of the performed epilepsy cases in North America and Europe remains disproportionally low, compared to the temporal lobe epilepsy cases [14, 15]. This underutilization of epilepsy surgery may be related to the fact that many patients, and their referring physicians, face with a lot of skepticism of a possible surgical intervention, due to the associated complications.

Indeed, temporal epilepsy surgery may be associated with complications as any other neurosurgical procedure. The occurrence of a temporal epilepsy surgery-associated neurological complication becomes even more dramatic, since the vast majority of the surgical candidates are neurologically intact. The resection or the disconnection of theoretically normal brain tissue, which is part of epilepsy surgery strategy, may lead to a behavioral and/or cognitive deficit [16]. Therefore, accurate knowledge of all potential complications of temporal lobe epilepsy surgery is of paramount importance for preventing them, if possible, or appropriately managing them when they occur. Furthermore, a well and accurately informed surgical candidate may rationally face the risks of any surgical interventions and properly weight them against the risks of no surgical intervention. Moreover, understanding of the underlying mechanisms of many of these complications may eventually lead to the development of imaging, neurophysiological, or surgical techniques, which may prevent their occurrence. 

Although the published number of temporal epilepsy surgery series is geometrically increasing, the number of publications regarding surgical or other procedure-related complications remains quite limited. This may be related to the fact that for several years many of the temporal epilepsy surgery-associated complications were considered as expected, inevitable, or acceptable complications [17]. Additionally, the existence of various classification schemes regarding complications and the utilization of different terms (complication or side effect) make the interpretation of the reported complication rates extremely difficult. Furthermore, the existing confusion along with the heterogeneity of the reported series makes almost impossible their comparison regarding the complication rates.

In our current study, we attempted to systematically review the existent literature regarding the temporal lobe epilepsy surgery associated complications and to identify, whenever possible, their underlying pathophysiologic mechanisms. We also attempted to summate all techniques and strategies reported in different surgical series, for providing a guide for preventing or minimizing the incidence of any temporal lobe resective surgery-related complications.

## 2. Material and Methods

An extensive literature search was performed in the PubMed medical database. The following terms were used for our search: “behavioral,” “cognitive,” “complication,” “deficit,” “disorder,” “epilepsy,” “hemianopia,” “hemianopsia,” “hemorrhage,” “lobectomy,” “medial,” “memory,” “mesial,” “neurobehavioral,” “neurocognitive,” “neuropsychological,” “psychological,” “psychiatric,” “quadranopia,” “quadranopsia,” “resective,” “side effect,” “surgery,” “temporal,” “temporal lobe,” and “visual field,” in any possible combination. Our search was limited within the last 23 years (1990–present). Furthermore, only papers published in English language and published in peer-reviewed journals were considered. All the retrieved titles and abstracts were meticulously reviewed. In addition, the reference lists from the retrieved papers were carefully reviewed to identify any additional pertinent papers for inclusion. Case reports were excluded from our current study. Moreover, complications associated with invasive preoperative monitoring were excluded from our current study, since the target of our analysis was solely resective-surgery associated complications.

Every possible effort was made to identify any repetition of cases among the published surgical series and any overlaps of series reported in different journals. In such occasions, only the original or the largest, regarding the number of participants, surgical series were included in our study. It has to be mentioned, however, that despite our efforts in identifying such repetitions, this task was not easy and the reader must be aware of potential redundancies in the reported data.

We attempted in our review to separate pediatric from adult surgical series. Frequently, the pediatric series included adolescents (12–18 yr), while other series had purely pediatric (≤12 yr) populations. In a few rare occasions, the reported surgical series included mixed, pediatric, and adult populations, without providing data regarding the percentage of each component. In these series that mixed populations are included, this is clearly indicated in our current study. Furthermore, the reported complications were grouped as surgical, neurological, and neuropsychological for presentation purposes, and a percentage was calculated whenever the total number of patients included in the surgical series was available. Additionally, a cumulative complication rate was calculated by adding the number of all complicated cases in each surgical series.

Unfortunately, temporal lobe resective surgery for epilepsy is an extremely wide term, describing a spectrum of surgical procedures and varying from simple neocortical lesionectomy to extensive anterior temporal lobectomy (ATL) and ipsilateral amygdalohippocampectomy (AH). In a large number of the reviewed surgical series there was a single surgical procedure performed and its associated complications were reported. However, in a few occasions there were various surgical techniques utilized, targeting the neocortex of the temporal lobe or the mesial temporal structures, or both of them. We took into consideration this variability and we provided this information whenever available. In a large number of the published surgical series, there were mixed data regarding temporal and extratemporal resective procedures, or temporal resective procedures, hemispherectomies, and corpus callosotomies. In these series that data for distinct groups were provided, the temporal lobe resective procedures and their complication rates were calculated, while those series, in which data from temporal and extratemporal surgical procedures were mixed, we excluded them from our study. Every effort was made to summate and present every procedure-associated complication, without characterizing them as major or minor, since such classification is not unanimously accepted. We also attempted to include in our report the permanent or transient character of each of the reported complications, whenever this information was provided in the published paper.

## 3. Results

Different combinations of the utilized search terms provided a total of 1681 papers, of which the combination of “temporal lobe epilepsy surgery complications” provided the largest number of papers (1325 abstracts), while the search “temporal lobe epilepsy surgery hemianopia” provided the smallest number of retrieved papers (26 abstracts). We meticulously reviewed all the retrieved abstracts and we carefully selected 55 papers reporting on temporal lobe surgical series complications. Subsequently, the reference lists of all the retrieved papers were meticulously reviewed and were crossed checked for any additional pertinent papers. Finally, a total of 58 full papers met our inclusion criteria and were analyzed in our current study.

### 3.1. Pediatric Surgical Series

All the retrieved pediatric surgical series reported no procedure-related deaths [6, 9, 13, 18–21]. The observed cumulative morbidity rates varied between 0–9.3% in the reviewed series [6, 9, 13, 18–21] (Table 1).

#### 3.1.1. Neurological Complications

Analysis of the reported complications confirms that the most common neurological complication is the development of postoperative visual field deficit (VFD) [6, 9, 18, 20]. Kim et al. [9] found that the incidence of postoperative superior quadranopsia in their cohort was 22.0%. However, Lopez-Gonzalez et al. [6] reported a significantly lower (1.5%) incidence of postoperative VFDs. Likewise, Erba et al. [18] reported postoperative hemianopsia in 4.3% of their patients and Terra-Bustamante et al. [20] in 2.9% of their series. Interestingly, Sinclair et al. [19] and Vadera et al. [13] reported no postoperative VFDs.

Postoperative hemiparesis occurred in 4.3% of the patients reported by Erba et al. [18], while the respective rate was 8.5% in the series reported by Kim et al. [9]. Transient speech difficulties occurred in 0.7% of the patients reported by Lopez-Gonzalez et al. [6], while Sinclair et al. [19] reported the development of postoperative stroke in 3.1% of their series. Erba et al. documented transient ipsilateral oculomotor nerve palsy in 2.1% of their patients [18].

#### 3.1.2. Surgical Complications

Postoperative hydrocephalus requiring surgical management occurred in 3.1% of the patients reported by Sinclair et al. [19]. Postoperative meningitis and/or surgical wound infections seemed to not be major problems in the reported series. Sinclair et al. [19] found postoperative bone flap infections in 3.1% of their patients, Erba et al. [18] reported superficial wound infections in 2.1% of their cohort, while Lopez-Gonzalez et al. [6] reported wound infection in 1.5% of their patients. Kim et al. reported slightly higher (8.5%) wound infection rate and meningitis rate of 3.4% [9]. However, in all the reported series infections were successfully managed with antibiotic administration, with no further sequelae.

#### 3.1.3. Neuropsychological Complications

The development of cognitive, behavioral, and/or psychiatric postoperative symptoms represents another procedure-related complication, even among children [6, 9, 18]. Erba et al. found that 4.3% of their patients developed postoperatively a syndrome of hypergraphia, hyperreligiosity, and sticky personality, while another 4.3% developed postoperative depression with suicidal ideation [18]. Similarly, Kim et al. [9] documented the development of postoperative psychosis in 5.1% of their patients, while Lopez-Gonzalez et al. [6] found that 10% of their patients developed postoperative *de novo* depression.

### 3.2. Mixed Population (Pediatric and Adult) Surgical Series

There are a few surgical series reporting on the complication rate of temporal resections in adult and pediatric patients [16, 22, 23] (Table 2). There were no deaths in the reported series, with the exception of Lee et al. who reported 3.5% mortality rate in their series [22]. They reported one death after developing severe hypoxic brain damage postoperatively caused most probably, according to the authors, by the induced hypoventilation for treating severe postoperative headache [22]. The cumulative complication rates including neurological, surgical, neuropsychological, and psychiatric complications varied between 3.8% and 26.1% [16, 22, 23]. 

#### 3.2.1. Neurological Complications

Salanova et al. reported that the most common complication in their series was the development of transient postoperative language difficulties, occurring in 3.7% of their patients [23]. The respective incidence of transient dysphasia (lasting less than 12 months), in the series reported by Tanriverdi et al. was 0.6% [16]. Salanova et al. [23] found that transient cranial nerve deficits occurred in 3.2% of their patients, while the respective rate reported by Tanriverdi et al. [16] was 0.3%. The incidence of postoperative hemiparesis ranged between 0.1% and 0.9% [16, 23]. Postoperative VFDs, hemianopsia, or superior quadranopsia were reported by Salanova et al. [23] in 0.4% of their patients and in 0.2% by Tanriverdi et al. [16].

#### 3.2.2. Surgical Complications

Surgical wound infection reported by Salanova et al. [23] in 1.3% of their patients, while Tanriverdi et al. [16] reported 0.5%, including cases of meningitis and cerebral abscess (0.3%). Tanriverdi et al. also reported the development of postoperative hematomas in 0.6% of their patients, postoperative transient brain edema in 0.2%, postoperative hydrocephalus in 0.1%, while 0.4% of their patients had subgaleal fluid collections and another 0.2% had problems with CSF leak through their surgical wound or skin suture detachment [16].

#### 3.2.3. Neuropsychological Complications

Salanova et al. found that the most common neuropsychological complication in their series was the development of postoperative verbal memory difficulties, in 8.8% of their patients [23]. They also noticed that 5.5% of their patients developed postoperatively depression, while another 2.3% developed postoperative psychosis [23]. Unfortunately, Tanriverdi et al. did not include in their detailed series their neuropsychological complications [16].

### 3.3. Adult Surgical Series

No deaths were reported in the retrieved adult surgical series [1, 4, 5, 15, 17, 24–30] (Table 3).

#### 3.3.1. Neurological Complications

The development of postoperative neurological deficits remains a major concern in epilepsy surgery. The occurrence of hemiparesis postoperatively has been reported to vary between 0 and 5%, with a wide range of severity, duration, and rehabilitation rates [4, 17, 21, 24, 26, 28, 30, 31]. This could be attributed either to the development of postoperative ischemia secondary to edema, ischemia secondary to cerebral vasospasm development, and excessive manipulation of the middle and/or dominant anterior choroidal arteries during resection [5, 17, 24, 28]. It has to be mentioned, that vasospasm has been detected by transcranial Doppler sonography in 32.7% of patients undergoing selective AH [32]. Engel et al. [30] reported that 21.4% of their patients demonstrated cerebral ischemic changes in their postoperative MRIs, although these were clinically significant in only 7.1% of their cases. 

 Dysphasic or more rarely aphasic postoperative symptomatology occurs in 1.7%–7.7% of the reported cohorts [4, 24, 26, 29, 31]. In the vast majority of cases, these symptoms are transient, and usually resolve within the first few postoperative weeks with no further consequences. However, in rare instances these deficits may be permanent. Falowski et al. [4] reported 0.9% incidence of permanent postoperative dysphasia, while Grivas et al. [26] found that the incidence of permanent dysphasia in their series (patients older than 50 yr) was 3.8%. 

 Cranial nerve deficits and mostly trochlear nerve palsy are usually responsible for the development of postoperative diplopia in patients undergoing ATL + AH. The incidence of trochlear nerve palsy has been reported to vary between 2.6% and 19% [17, 24, 26, 31, 33, 34]. In the vast majority of the reported cases, postoperative diplopia spontaneously resolved with no further sequelae [17, 24, 26, 31, 33, 34]. Contrariwise, the observed postoperative VFDs are permanent. Their incidence presents a high variation, with rates ranging from 1.8% to 69% [24, 26, 28, 33, 33, 34]. In the vast majority of the reported series, these VFDs were superior quadranopsia [23, 35–37]. However, Heller et al. [28] reported 1.8% incidence of complete contralateral homonymous hemianopsia, while Grivas et al. [26] found similar findings in 5.8% of their patients. It has to be mentioned that in several surgical series the exact incidence of VFDs is not available, since many epilepsy surgeons consider the occurrence of VFDs postoperatively inevitable [4, 17].

#### 3.3.2. Surgical Complications

The formation of postoperative hematomas, either intraparenchymal or epi-/subdural, was quite rare among the reported series. Sindou et al. reported postoperative hematoma in 3% of their patients [17]. These were hematomas of the resection cavity, which were developed within the first 24 postoperative hours. They had to surgically evacuate them, with no further sequelae for their patients [17]. Likewise, Grivas et al. reported 3.8% incidence of postoperative hematomas in a series of elderly (≥50 yr) patients undergoing AH or cortical lesionectomy and AH [26]. They had to surgically intervene and evacuate all these hematomas, with no further consequences [26]. Heller et al. reported 1.8% incidence of postoperative epidural hematoma, which required surgical evacuation, while another 1.8% of their patients developed postoperative hygromas [28]. Ipekdal et al. reported that 1.7% of their patients developed postoperative subdural effusion, which was spontaneously resolved [5]. An interesting finding was the development of immediate (within the first few hours) postoperative cerebellar hemorrhage in cases of ATL + AH [38, 39]. Toczek et al. [39] reported 4.9% incidence rate of cerebellar hemorrhage, while de Paola et al. [38] found that the occurrence of cerebellar hemorrhage was slightly lower (2.5%) in their cohort. Although the exact pathophysiologic mechanism responsible for this complication remains unclear, it has been postulated that undetected blood coagulation abnormalities, which may be present in epileptic patients with chronic use of anticonvulsant medications could be responsible for this distant hematoma formation [39]. The intraoperative drainage of large amounts of CSF during ATL, in association with the rapid volume change of intracranial compartments, could be another mechanism responsible for the postoperative development of distant hematomas [39–41]. A similar mechanism may well be responsible for the development of lumbar subdural hematomas in cases of ATL + AH [35]. It has been reported that these spinal subdural hematomas were developed after the third postoperative day and required no surgical intervention [35].

Sindou et al. reported that in 2% of their patients, hydrocephalus developed postoperatively, for which shunt insertion was necessary [17]. Engel et al. [30] reported 7.1% incidence of postoperative hydrocephalus in their cohort. Complications related to the skin incision and to the dissection of the soft tissues during craniotomy have been reported in many series [5, 15, 24]. Characteristically, Roberti et al. reported palsy of the frontal branch of the facial nerve in 2.4% of their patients, while another 2.3% experienced problems with bone flap reabsorption [15]. Likewise, Ipekdal et al. reported frontal branch injury and postoperative palsy in 1.7% of their patients [5]. Acar et al. reported similar rates of frontal branch palsy (2.6%), while in another 2.6% hemotympanum occurred secondary to opening of the air mastoid cells during the craniotomy [24].

Infections in the postoperative period represented either superficial wound infections or meningitis. The reported postoperative infection rates ranged between 1% and 4.7% [4, 5, 17, 28, 31]. Sindou et al. [17] reported 3% meningitis rate, while Heller et al. [28] found 1.8% cases of bacterial meningitis, and Ipekdal et al. [5] in 4.7% of their patients. However, in none of the reported cases antibiotic treatment of meningitis failed.

Other various procedure-related complications were also reported in the reviewed surgical series [4, 26, 28]. Heller et al. [28] reported deep venous thrombosis (DVT) in 1.8% of their patients, while Falowski et al. [4] found that DVT occurred in 0.9% of their cohort. Grivas et al. reported pneumonia in 1.9% and pulmonary embolism in another 1.9% of their patients [26].

#### 3.3.3. Neuropsychological Complications

Postoperative memory decline constitutes one of the most worrisome temporal resective surgery complications, particularly in cases of the dominant temporal lobe. Ojemann and Dodrill emphasized the negative effect of dominant hemisphere temporal lobectomy in the patient's verbal memory [42]. In their original series, they found 22% verbal memory decline at one month after surgery and 11% at the completion of the first postoperative year [36]. Gleissner et al. [43] reported their results from a series of adult patients, who underwent detailed verbal and visual memory evaluations before and then at 3 months postoperatively. They found that there was 51% loss of verbal memory in left-sided cases, while the respective percentage among right-sided resections was 32%. The postoperative visual memory loss was approximately 27% in their series. They noticed that the verbal memory loss was more frequent among males [43]. Comparable memory loss rates were documented by Grivas et al. in their series [26]. They found postoperative verbal memory decline in 29.4%, while the visual memory loss was 32.4% in their patients [26]. Acar et al. [24] reported 5.1% memory deficits in their series, while Helmstaedter et al. [12] found that there was significant postoperative verbal learning recognition deficit in their left-sided lobe resections. Similarly, Bell et al. [2] found significant postoperative loss of verbal ability in their patients undergoing left-sided ATL + AH for nonlesional epilepsy.

During the last two decades, the number of published series examining the development of *de novo* postoperative psychiatric entities or investigating the role of temporal resective surgery in the exacerbation of preoperative psychiatric conditions has been exponentially growing [5, 26, 44–53]. The reported psychiatric clinical entities include psychosis, depression, anxiety disorders, and obsessive-compulsive disorders [5, 26, 44–53]. Older reports stated that the actual incidence of postoperative psychosis was approximately 8%, which was at the lower edge of the previously published series [54]. Indeed, Leinonen et al. [48] reported that *de novo* psychosis occurred in 5.3% of their series of patients undergoing temporal lobectomy. Grivas et al. [26] reported 5.8% incidence of postoperative organic psychological syndrome. Shaw et al. [51], in a recent series, found that the incidence of *de novo* psychosis among patients undergoing ATL + AH was 3.4%. They also found that there was a clustering of psychosis development within the first 12 postoperative months. Christodoulou et al. [45] reported even lower incidence of postoperative psychosis in their series (1.1%), while Malmgren et al. [49] found 0.5% incidence of *de novo* psychosis and Ipekdal et al. [5] reported 1.7% incidence of *de novo* psychosis. However, it has to be emphasized that temporal resections may exacerbate preexisting psychosis [44, 46, 49]. Blumer et al. [44] found worsening of the pre-existing psychiatric conditions in 24% of their patients.

Naylor et al. [50] found 10.8% of postoperative depression in their series. They reported that the vast majority of these were *de novo* cases (8%), while the remaining (2.8%) represented worsening of a pre-existing condition. Blumer et al. [44] found that in their cohort, 42.1% of their preoperatively intact from psychiatric standpoint patients developed depression after temporal lobectomy. Wrench et al. [53] reported 10% incidence of *de novo* postoperative depression. However, Ipekdal et al. [5] found significantly lower rates (1.7%) of postoperative depression in their series. Malmgren et al. [49] found that the incidence of anxiety disorders increased from 1.4% preoperatively to 17% postoperatively. Likewise, the occurrence of affective disorders in their series increased from 1.4% before surgery to 17% after surgery. Ipekdal et al. [5] found postoperative anxiety disorders in 1.7% of their patients, while Kulaksizoglu et al. [47] reported the postoperative development of *de novo* obsessive-compulsive disorders in patients undergoing temporal resections.

## 4. Discussion

Reviewing of complications associated with resective temporal surgery for epilepsy is a quite complicated issue. This is related to the fact that the reported series used different surgical techniques, with or without neocortical resection, with different extent of hippocampal resection, and with varying anatomical avenues for resecting the mesial temporal structures. The underlying temporal lobe pathology also varied significantly among the published series, while the patients' groups of the published series are far from being considered homogenous and comparable. Occasionally, even in the same series there is utilization of different surgical techniques and/or participation of more than one epilepsy surgeon. Thus, comparison between the reported complication rates is almost impossible. In addition, many of the commonly observed complications (visual field deficits, cognitive, and psychiatric postoperative changes) of temporal lobectomy are underreported, since for a long period of time were considered from many epilepsy surgeons as either inevitable complications or acceptable side effects and, therefore, were excluded from their complication reports. However, detailed and accurate knowledge of the potential complications is of paramount importance for appropriately informing the surgical candidates during the decisionmaking process for avoiding complications' development if possible and for identifying them in a timely fashion and properly managing them.

Moreover, the published rates of certain complications may be greatly affected by the utilized method for detecting them. The detection of a postoperative superior quadranopsia may be undetected during a confrontational clinical visual field examination. The employment of official visual field examination may increase the incidence of postoperative VFDs. Even the employment of different VF examination methodologies may provide different rates of VFDs [36, 55]. Manji and Plant [36] reported 47% incidence of VFD when their patients were examined with the Eastman method, while VFDs were documented in 54% of the patients in the same series when the Goldman perimeter was used.

Similarly, the actual incidence of postoperative cognitive deficits may be higher when detailed pre- and postoperative cognitive evaluations will be employed. This may well explain the observed variation between the reported postoperative memory decline rates among the different series. Another confounding factor in determining the accuracy of the reported memory decline rates may be the exact time of postoperative cognitive evaluation. The adaptation of a widely accepted cognitive pre- and postoperative evaluation methodology could provide comparable results among different series and a more realistic estimate about the true incidence of memory decline. Similar inaccuracies are applicable in regard to the incidence of postoperative psychiatric complications. Although, many reports have pointed out the occurrence of psychosis, depression, and anxiety disorders after temporal lobectomy, their actual incidence remains still controversial [44–47, 49–53, 56–62]. The differentiation of *de novo* from relapsing, preexisting, or preoperatively undetected cases remains a major problem, in determining the exact rate of postoperative psychiatric complications. Detailed pre- and postoperative psychiatric evaluation of all surgical candidates may define the true occurrence of postoperative psychopathology and improve the patients' surgical outcome and their quality of life.

Several parameters have been identified by various clinical investigators in the reported temporal lobectomy series as predisposing factors for complications' development. The patient's age has been recognized as an important factor in the temporal lobectomy-associated morbidity [21, 23, 26]. This may be attributed to the employment of preoperative invasive monitoring more frequently in pediatric patients or may be related to the patients' age [21]. Younger adult patients seemed to have lower complication rates than older adults in several of the reported series [23, 26]. Contrariwise, Sindou et al. [17] found no significant correlation between their complication rate and the patient's age, the side, or the extent of temporal resection. Heller et al. [28] identified in their study the surgeon's experience as an important factor in the development of complications. Oertel et al. [31] identified the utilization of a neuronavigational system as a factor decreasing their complication rate. Furthermore, the exact role of structural lesions versus nonlesional temporal cases in the development of postoperative complications remains to be defined.

The development of cognitive, neuropsychological, and/or psychiatric complications has been associated with various factors [2, 43, 46, 49, 51, 53]. Gleissner et al. [43] found that postoperative loss of visual memory decline was associated with the patient's gender and the laterality of resection (patients with left-sided resections had more commonly verbal memory loss). The positive history for anxiety and/or depression disorders, the positive family history for psychiatric disorders, the presence of mood disturbances preoperatively, the postoperative continuation of seizures, the difficulty in postoperative psychosocial adjustment, the diffuse epileptogenicity, and the presence of secondary generalized tonic-clonic seizures preoperativel have all been identified as predisposing factors for developing postoperative psychiatric complications [46, 49, 53]. Shaw et al. [51] have demonstrated that patients with histopathology other than mesial temporal sclerosis, the presence of bilateral EEG abnormalities, and the small size of the contralateral amygdala may all predispose to postoperative psychosis development. However, there are reports implicating the large size of the contralateral to the resection amygdala in the pathogenesis of postoperative psychosis [52]. The exact role of the side of resection, the extent of the mesial and neocortical temporal resection, the underlying histopathology, and the presence of previous temporal surgeries in the development of postoperative cognitive and psychiatric complications remains to be accurately defined in the future.

The development of newer imaging modalities and the wider application of intraoperative electrophysiological methodologies may contribute in preventing or minimizing the possibility of VFDs after temporal resective surgery [37, 63, 64]. The employment of preoperative advanced MR imaging techniques, such as diffusion tensor imaging (DTI) and fiber tracking (FT), may help in outlining the visual tracts [37, 63, 64]. Registration of the DTI data on the preoperative neuronavigational planning may protect the visual pathway, without compromising though the extent of the resection. Additionally, the intraoperative employment of visual evoked potential monitoring and application of direct subcortical electrical stimulation may further increase the accuracy of resection along the visual pathway and thus may minimize the chance of postoperative VFDs. Similarly, the employment of careful dissection along the trochlear nerve and the avoidance of even gentle retraction on the nerve may prevent or minimize the incidence of postoperative diplopia [24, 33].

The occurrence of postoperative hemiparesis after temporal lobectomy has been attributed to the development of vasospasm of the middle cerebral or of the dominant anterior choroidal arteries or the development of cerebral edema secondary to surgical manipulation [26, 28, 65]. The selective employment of intraoperative micro-Doppler in high-risk patients may identify those who are predisposed to vasospasm development. The early pharmacological treatment of these patients with nimodipine could prevent the development of clinically symptomatic vasospasm and thus prevent the development of ischemic strokes and postoperative hemiparesis. Likewise, the issue of administering perioperative antibiotics for preventing any infections and the selection of the proper antibiotic prophylaxis regimen remains controversial. Although the vast majority of epilepsy surgeons administer perioperative antibiotic prophylaxis, there are reports questioning its role [16]. Surgical complications related to the skin incision or the craniotomy may be prevented by employing interfascial dissection for avoiding any injuries to the frontal branch of the facial nerve and thus accomplish a better postoperative cosmetic result [15].

## 5. Conclusions

Temporal resective surgery for medically refractory epilepsy constitutes an extremely safe procedure, with mortality rates approaching zero. The reported procedure-associated complications may be classified into three large groups: surgical, neurological, and neurocognitive/psychological. The most common surgical complications include infection (surgical wound, meningitis, and cerebral abscess), postoperative hematoma formation (resection cavity hematoma, intraparenchymal adjacent to the resective site, distant cerebral or spinal, or epi/subdural), palsy of the frontal branch of the facial nerve secondary to intraoperative injury, postoperative hydrocephalus, CSF leakage, and wound healing problems. Although these complications are rare, modification of the surgical technique by employing careful interfascial dissection during the craniotomy, meticulous hemostasis at the completion of the resection, and routine evaluation of the patient's coagulation profile preoratively, intraoperatively, and for the first 2-3 days postoperatively may further decrease their incidence.

The neurological complications include postoperative VFDs, hemiparesis, dysphasia/aphasia, cerebral ischemic changes, and cranial nerve paresis or palsy. More sensitive detection and more accurate documentation of VFDs are necessary for understanding their actual postoperative incidence. Application of advanced MR imaging techniques, such as DTI and FT, and intraoperative neurophysiologic monitoring may decrease the incidence of postoperative VFDs. The employment of intraoperative micro-Doppler and postoperative transcranial Doppler for early detection of cerebral vasospasm may prevent or minimize the incidence of postoperative ischemic events and thus the occurrence of postoperative hemiparesis. The importance of minimal and gentle manipulations of the adjacent vascular structures during resection cannot be overemphasized.

Postoperative cognitive and psychological deficits include decline of preoperative verbal and visual memory, as well as exacerbation of preexisting or *de novo* development of psychosis, depression, and anxiety disorders. Early diagnosis of postoperative psychological and cognitive disturbances may help in their more efficient management and may improve the patients' overall outcome. Finally, proper identification of any predisposing to psychopathology factors preoperatively may help in the early diagnosis of such psychiatric complications and may help in their prompt treatment.

## Figures and Tables

**Table 1:** Synopsis of data of pediatric temporal lobe epilepsy surgical series and their reported complications.

Series/year of publication	Study characteristics	Number of pts	Surgical procedure	Mortality	Mean followup	Seizure-free outcome	Complications	Cumulative complication rate*	Behavioral/cognitive/psychiatric complications
Erba et al., 1992 [18]	Prospective, pediatric & adolescent	46	Standard ATL + AH	0%	5 years	85%	Infection: 2.1% Hemiparesis/hemianopsia: 4.3% C.N. Palsy: 2.1%	17.1%	Depression: 4.3% Behavioral syndrome: 4.3%

Sinclair et al., 2003 [19]	Retrospective, pediatric	32	Standard ATL	0%	6.9 years	76%	Infection: 3.1% Hydrocephalus: 3.1% Stroke: 3.1%	9.3%	N/A

Terra-Bustamante et al., 2005 [20]	Prospective, pediatric & adolescent	35	Standard ATL + AH	0%	3.5 years	77.1%	Hemianopsia: 2.9%	2.9%	N/A

Kim et al., 2008 [9]	Prospective, pediatric	59	Standard ATL + AH	0%	62.3 months	69%	VFDs: 22.0% Hemiparesis: 8.5% Wound infection: 8.5% Meningitis: 3.4%	47.5%	Psychosis: 5.1%

Lopez-Gonzalez et al., 2012 [6]	Retrospective, pediatric	130	Cortico-amygdalo-hippocampectomy (CAH), Lesionectomy + CAH, Lesionectomy, neocortical resection, tailored ATL + AH, selective AH	0%	2 years	72%	VFDs: 1.5% Transient speech Difficulties: 0.7% Infection: 1.5%	17%	Depression: 10%

Vadera et al., 2012 [13]	Retrospective, pediatric	45	Standard ATL + AH	0%	60.2 months	69%	None	0%	N/A

*This rate includes the behavioral/cognitive/psychiatric complications. ATL: anterior temporal lobectomy; AH: amygdalohippocampectomy; VFDs: visual field deficits; C.N.: cranial nerve.

**Table 2:** Synopsis of data of mixed pediatric and adult temporal lobe epilepsy surgical series and their reported surgical complications.

Series/year of publication	Study characteristics	Number of pts	Surgical procedure	Mortality	Mean followup	Seizure-free outcome	Complications	Cumulative complication rate*	Behavioral/ cognitive/ psychiatric complications
Salanova et al., 2002 [23]	Prospective	215	Temporal resective surgery	0%	7 years	69%	Dysphasia: 3.7% Hemiparesis: 0.9% C.N. palsy: 3.2% VFDs: 0.4% Infection: 1.3%	26.1%	Verbal memory deficits: 8.8% Depression: 5.5% Psychosis: 2.3%

Lee et al., 2008 [22]	Retrospective	28	ATL + AH	3.5%	N/A	N/A	N/A	10.6%	N/A

Tanriverdi et al., 2009 [16]	Retrospective	1232	ATL + AH, Selective AH	0%	At least 1 year	N/A	Dysphasia: 0.6% Hemiparesis: 0.1% C.N. palsy: 0.3% VFDs: 0.2% Infection: 0.5% Hematomas: 0.6% Hydrocephalus: 0.1% Subgaleal Collections: 0.4% CSF Leakage: 0.1% Suture Detachment: 0.1%	3.8%	N/A

*This rate includes the behavioral/cognitive/psychiatric complications. ATL: anterior temporal lobectomy; AH: amygdalohippocampectomy; VFDs: visual field deficits; C.N.: cranial nerve.

**Table 3:** Synopsis of data of adult temporal lobe epilepsy surgical series and their reported surgical complications.

Series/year of publication	Study characteristics	Number of pts	Surgical procedure	Mortality	Mean followup	Seizure-free outcome	Complications	Cumulative complication rate*	Behavioral/ cognitive/psychiatric complications
Wiebe et al., 2001 [1]	Prospective	40	ATL + AH	0%	12 months	54%	None	5%	5% memory deficits

Cohen-Gadol et al., 2003 [33]	Prospective	47	ATL + AH	0%	6 months	N/A	Diplopia: 19% VFDs: 69%	88%	N/A

Oertel et al., 2004 [31]	Prospective	60	Transcortical selective AH	0%	N/A	N/A	Hemiparesis: 5% Aphasia: 1.7% C.N. palsy: 5% Infections: 1.7%	13.4%	N/A

Grivas et al., 2006 [26]	Retrospective	52	AH, lesionectomy + AH	0%	33 months	71%	Hemiparesis: 3.8% Aphasia: 3.8% C.N. palsy: 5.8% VFDs: 5.8% Hematomas: 3.8% Pneumonia: 1.9% Pulmonary embolism: 1.9%	53.4%	Decline in neuropsychological status: 26.5% (Verbal memory decline in 29.4%, visual memory decline in 32.4%)

Sindou et al., 2006 [17]	Retrospective	100	Tailored ATL + AH	0%	53 months	85%	Hemiparesis: 2% C.N. palsy: 5% Hydrocephalus: 2% Meningitis: 3% Hematomas: 3% Pulmonary embolism: 1%	19%	Permanent neuropsychological impairment: 3%

Roberti et al., 2007 [15]	Retrospective	42	Tailored ATL + AH	0%	60 months	64%	Frontal branch of facial n. palsy: 2.4% Bone flap reabsorption: 2.3%	4.7%	N/A

Acar et al., 2008 [24]	Retrospective	39	Transcortical selective AH	0%	25.9 months	82%	Hemiparesis: 2.6% Dysphasia: 7.7% VFDs: 10.3% C.N. palsy: 2.6% Hemotympanum: 2.6% Frontal branch of facial n. palsy: 2.6%	33.5%	Memory difficulties: 5.1%

Elsharkawy et al., 2009 [25]	Retrospective	483	ATL + AH	0%	2 years	72.3%	Permanent deficits: 2.3% Temporary deficits: 2.3%	5%	N/A

Heller et al., 2009 [28]	Retrospective	55	Tailored ATL + AH	0%	N/A	N/A	Hemiparesis: 1.8% Stroke: 5.5% VFDs: 1.8% Hematomas/hygromas: 3.6% Meningitis: 1.8% Deep venous thrombosis: 1.8%	16.3%	N/A

Ipekdal et al., 2011 [5]	Retrospective	58	ATL + AH	0%	N/A	N/A	Stroke: 1.7% Frontal branch of Facial n. palsy: 1.7% Infections: 4.7% Subdural effusion: 1.7%	14.9%	Psychosis: 1.7% Depression: 1.7% Anxiety disorders: 1.7%

Falowski et al., 2012 [4]	Retrospective	222	Tailored AH	0%	5.4 years	70%	Dysphasia: 0.9% Infections: 1.4% Deep venous thrombosis: 0.9%	3.2%	N/A

*This rate includes the behavioral/cognitive/psychiatric complications. ATL: anterior temporal lobectomy; AH: amygdalohippocampectomy; VFDs: visual field deficits; C.N.: cranial nerve.
